# Bisphenol S causes excessive estrogen synthesis by activating FSHR and the downstream cAMP/PKA signaling pathway

**DOI:** 10.1038/s42003-024-06449-2

**Published:** 2024-07-10

**Authors:** Xiaorong Zhang, Xinda Zhang, Zhenzhong Zhang, Yijiao Shi, Jun Wang, Shaoguo Ru, Hua Tian

**Affiliations:** 1 College of Marine Life Sciences, Ocean University of China, 266003 Qingdao, Shandong Province China; 2 Tai’an Agriculture and Rural Affairs Bureau, 271000 Tai’an, Shandong Province China

**Keywords:** Pathogenesis, Endocrinology

## Abstract

Estrogen excess in females has been linked to a diverse array of chronic and acute diseases. Emerging research shows that exposure to estrogen-like compounds such as bisphenol S leads to increases in 17*β*-estradiol levels, but the mechanism of action is unclear. The aim of this study was to reveal the underlying signaling pathway-mediated mechanisms, target site and target molecule of action of bisphenol S causing excessive estrogen synthesis. Human ovarian granulosa cells SVOG were exposed to bisphenol S at environmentally relevant concentrations (1 μg/L, 10 μg/L, and 100 μg/L) for 48 h. The results confirms that bisphenol S accumulates mainly on the cell membrane, binds to follicle stimulating hormone receptor (FSHR) located on the cell membrane, and subsequently activates the downstream cyclic adenosine monophosphate/protein kinase A (cAMP/PKA) signaling pathway, leading to enhanced conversion of testosterone to 17*β*-estradiol. This study deepens our knowledge of the mechanisms of environmental factors in pathogenesis of hyperestrogenism.

## Abstract (web summary)

This study deepens the knowledge of mechanisms of environmental factors in pathogenesis of hyperestrogenism, which is indispensable for the prevention and treatment of hyperestrogenism and a diverse array of hyper-estrogen activity-driven diseases.

## Introduction

Estrogen not only controls almost all aspects of reproductive health in females and even in males, but is also indispensable to bone health, glucose homeostasis, immune robustness, cardiovascular health, and neural functions^1,2^. Too low or excessive levels of estrogen have been linked to a diverse array of chronic and acute diseases^2^. For example, estrogen excess in females has been linked to breast hypertrophy, short stature, autoimmune diseases such as systemic lupus erythromatosus and multiple sclerosis and several forms of cancer including breast, gastric, lung, hepatic, pituitary, and thyroid cancers^2^. Although heredity is an important cause of hyperestrogenism, the unprecedented escalation in epidemic of these hyper-estrogen activity-driven diseases indicates that other factor(s) are involved^2^. Emerging research shows that exposure to estrogen-like endocrine disrupting chemicals (EDCs) leads to increases in 17*β*-estradiol (E_2_) levels^3–9^.

Bisphenols are the most classic estrogen-like EDCs^10^. Considering the estrogen-like effects of bisphenol A (BPA), several countries and international organizations have proposed regulations to restrict its use, and accordingly bisphenol S (BPS), as a main substitute for bisphenol A, is widely used in the production of polycarbonate plastics, epoxy resins and consumer products including food containers, paper products, toys, medical equipment and electronics^11,12^. Due to widespread human exposure occurring *via* dietary^13^, inhalation^14^, and dermal contact^15^, BPS was reported in human serum^16^, urine^17^, amniotic fluid^14^, cord blood^18^, and breast milk^19^. For example, monitoring data from the National Health and Nutrition Examination Survey (NHANES) (2013-2014) showed that BPS was detected in 89.4% of randomly selected urine samples, suggesting that exposure to BPS is ubiquitous in the general U. S. population^20^. BPS was detected in human serum with the highest concentrations in samples from Nanjing (median: 0.65 ng/mL, maximum: 169 ng/mL) among the four cities studied, Wuhan, Huangshi, Nanjing, and Zhenjiang^16^. Unfortunately, evidence from both in vivo and in vitro studies indicates that BPS also possesses estrogen-like effects, increasing E_2_ levels in serum of female mouse/rats^3,4^, plasma of female zebrafish^5–7^, ovine granulosa cells^8^ and bovine granulosa cells^9^. However, the mechanism of action of BPS increasing estrogen synthesis is unknown, and further studies are urgently needed to fill this knowledge gap.

In females, estrogen is mainly produced by ovarian granulosa cells under the influence of pituitary-derived follicle-stimulating hormone (FSH)^1,21,22^, which stimulates gene expression of *CYP19A1* (the gene encoding CYP19A1, a key enzyme responsible for the conversion of testosterone (T) to E_2_) *via* binding to follicle-stimulating hormone receptor (FSHR) on the cytomembrane and then activating the downstream cyclic adenosine monophosphate (cAMP)/protein kinase A (PKA) and Ca^2+^/protein kinase C (PKC) signaling pathways^23–25^. The aim of this study was to reveal the underlying signaling pathway-mediated mechanisms, target site, and target molecule of action of BPS causing excessive estrogen synthesis. First, effects of BPS exposure at environmentally relevant concentrations (1 μg/L, 10 μg/L, and 100 μg/L) for 48 h on estrogen synthesis in human ovarian granulosa cells SVOG were evaluated by T content, E_2_ content, E_2_/T ratio, CYP19A1 enzyme activities, CYP19A1 protein expression, *CYP19A1* gene expression and width of endoplasmic reticulum. Then, the response of cAMP/PKA and Ca^2+^/PKC signaling pathways to BPS exposure was examined, and combined exposure of BPS and PKA inhibitor H-89 was carried out, to clarify the role of this signal cascade in mediating the effects of BPS on stimulating estrogen synthesis. Finally, the subcellular distribution of BPS was examined, BPS-bovine serum albumin (BSA) coupling exposure assay was employed, the expression of FSHR on the cell membrane was detected, the interaction between BPS and FSHR was determined, and combined exposure of BPS and FSHR antagonist hFSH-*β*-(33-53) was conducted, to clarify the target site and target molecule of action of BPS.

## Results

### No significant cytotoxicity of BPS was observed

Relative cell proliferation was not influenced after BPS exposure for 48 h at concentrations of 1 μg/L, 10 μg/L and 100 μg/L (*P* > 0.05) (Fig. S1). Thus, data in this paper are effects observed at non-cytotoxic concentrations.

### BPS exposure caused excessive estrogen synthesis

**Fig. 1:**
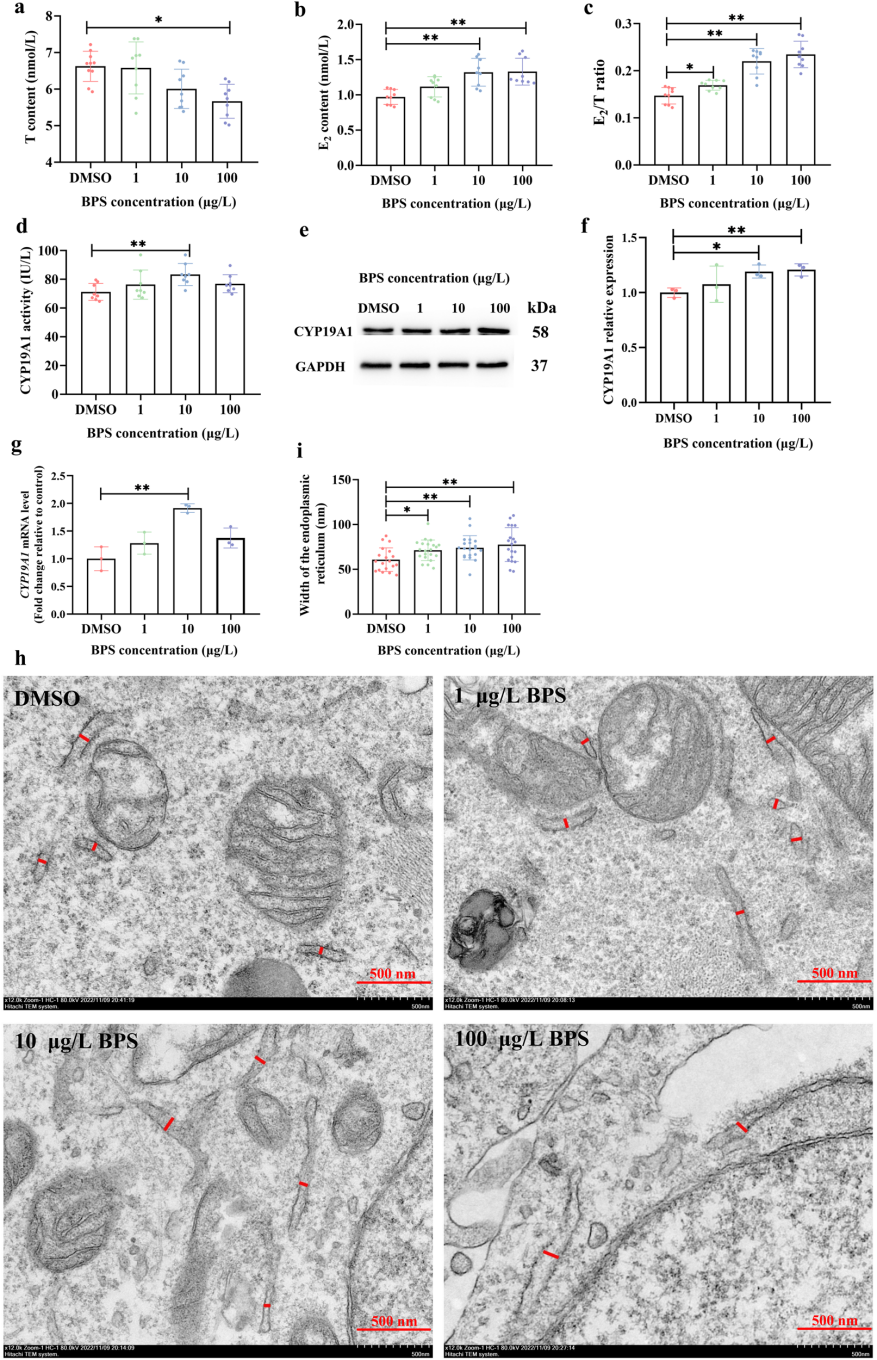
BPS exposure for 48 h caused excessive estrogen synthesis in SVOG cells. **a** T content (*n* = 9). **b** E_2_ content (*n* = 9). **c** E_2_/T ratio (*n* = 9). **d** CYP19A1 activity (n = 8). **e** Representative images of Western blotting for relative expression of CYP19A1. **f** Densitometric analysis of Western blotting for relative expression of CYP19A1 (*n* = 3) (**g**) *CYP19A1* gene expression (*n* = 3). **h** Representative images of transmission electron microscope. The red line indicates width of endoplasmic reticulum, and the scale bar is 500 nm. **i** Quantitative analysis of width of endoplasmic reticulum (n = 21). Data are expressed as mean ± SD. * indicated a significant difference from the solvent control (0.01 < *P* < 0.05), and ** indicated a highly significant difference from the solvent control (*P* < 0.01).

BPS exposure at a concentration of 10 μg/L upregulated E_2_ content (*P* < 0.01) (Fig. 1b), CYP19A1 enzyme activities (*P* < 0.01) (Fig. 1d), CYP19A1 protein expression (*P* < 0.05) (Fig. 1e–f) and *CYP19A1* gene expression (*P* < 0.01) (Fig. 1g). BPS exposure at a concentration of 100 μg/L downregulated T content (0.01 < *P* < 0.05) (Fig. 1a) while upregulated E_2_ content (*P* < 0.01) (Fig. 1b) and CYP19A1 protein expression (*P* < 0.01) (Fig. 1e–f). 1 μg/L, 10 μg/L and 100 μg/L BPS exposure caused upregulation of E_2_/T ratio (*P* < 0.01 or 0.01 < *P* < 0.05) (Fig. 1c) accompanied by widening of endoplasmic reticulum (*P* < 0.01 or 0.01 < *P* < 0.05) (Fig. 1h-i).

Under the condition of supplementation with T, E_2_ levels were still upregulated by BPS exposure at concentration of 10 μg/L and 100 μg/L (*P* < 0.01) (Fig. S2a).

### BPS exposure caused excessive estrogen synthesis through the cAMP/PKA signaling pathway

**Fig. 2:**
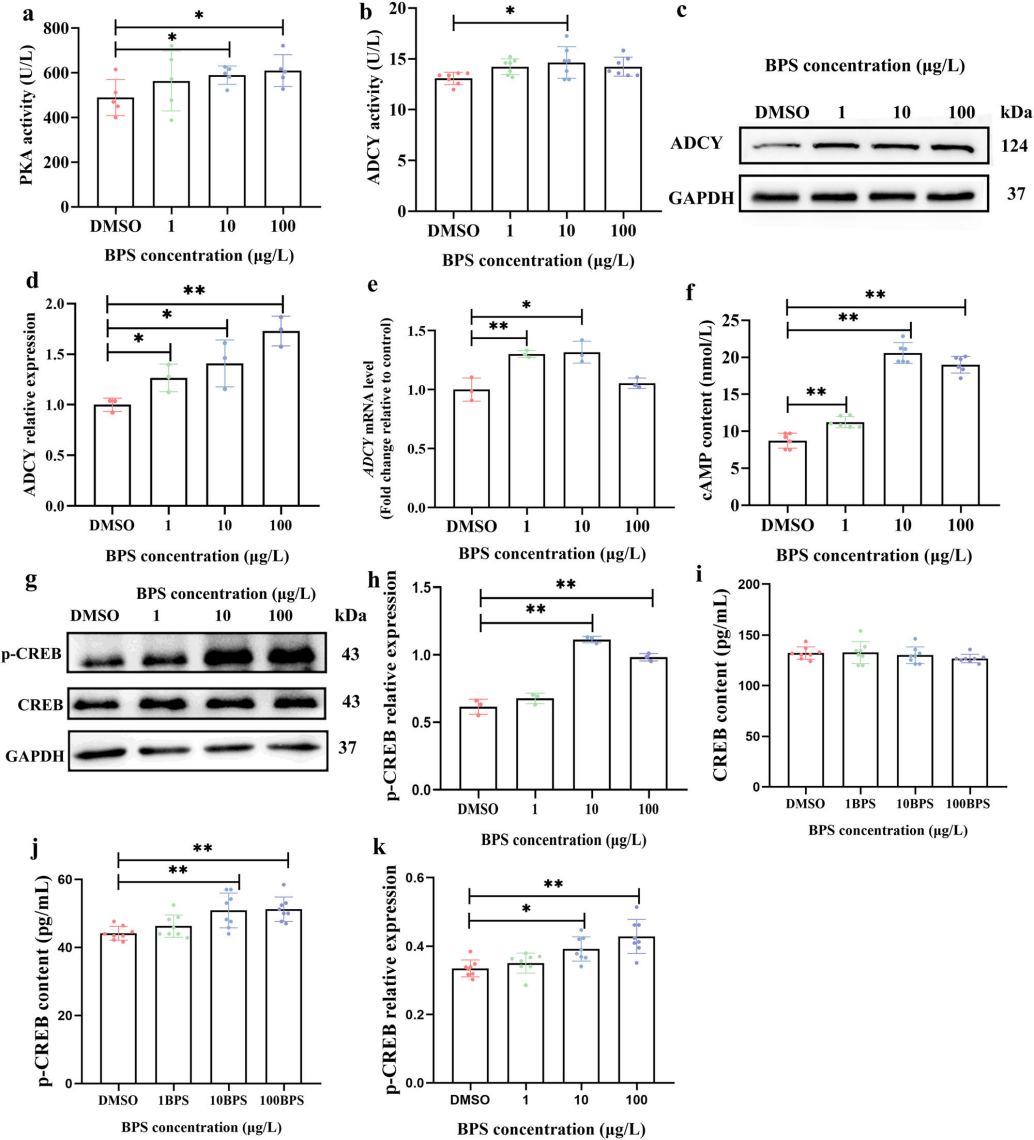
BPS exposure for 48 h upregulated cAMP/PKA signaling pathway in SVOG cells. **a** PKA activity (*n* = 5). **b** ADCY activity (*n* = 7). **c** Representative images of Western blotting for relative expression of ADCY. **d** Densitometric analysis of Western blotting for relative expression of ADCY (*n* = 3). **e** *ADCY* gene expression (*n* = 3). **f** cAMP content (*n* = 6). **g** Representative images of Western blotting for relative expression of p-CREB. **h** Densitometric analysis of Western blotting for relative expression of p-CREB (*n* = 3). **i** CREB concentration detected by ELISA (*n* = 8). **j** p-CREB concentration detected by ELISA (*n* = 8). **k** Relative expression of p-CREB detected by ELISA (*n* = 8). Data are expressed as mean ± SD. * indicated a significant difference from the solvent control (0.01 < *P* < 0.05), and ** indicated a highly significant difference from the solvent control (*P* < 0.01).

BPS exposure at 10 μg/L and 100 μg/L upregulated PKA activities (0.01 < *P* < 0.05) (Fig. 2a), while PKC activities were not influenced (*P* > 0.05) (Fig. S3). Other cAMP/PKA signaling pathway-related indicators including ADCY activities (0.01 < *P* < 0.05) (Fig. 2b), ADCY protein expression (*P* < 0.01 or 0.01 < *P* < 0.05) (Fig. 2c-d), *ADCY* gene expression (*P* < 0.01 or 0.01 < *P* < 0.05) (Fig. 2e) and cAMP content (*P* < 0.01) (Fig. 2f) were also upregulated by BPS exposure, with exceptions of ADCY activities at 1 μg/L and 100 μg/L BPS exposure groups (*P* > 0.05) (Fig. 2b) and *ADCY* gene expression at 100 μg/L BPS exposure group (*P* > 0.05) (Fig. 2e). Besides, both the results of Western blotting (Fig. 2g, h) and enzyme-linked immunosorbent assay (ELISA) (Fig. 2i–k) showed that BPS exposure at 10 μg/L and 100 μg/L increased cAMP-response element binding protein (CREB) phosphorylation levels (*P* < 0.01 or 0.01 < *P* < 0.05).

**Fig. 3:**
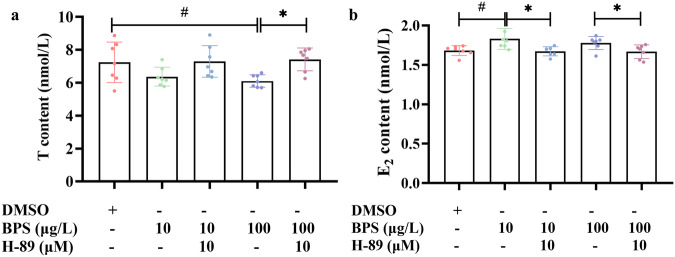
Combined exposure to BPS and PKA inhibitor H-89 restored estrogen synthesis to levels comparable to those of the solvent control in SVOG cells. **a** T content. **b** E_2_ content. *n* = 7. Data are expressed as mean ± SD. ^#^ indicated a significant difference from the solvent control (0.01 < *P* < 0.05), and * indicated a significant difference between the combined exposure group to BPS and H-89 and the BPS single exposure group (0.01 < *P* < 0.05).

Furthermore, combined exposure to BPS (10 μg/L and 100 μg/L) and PKA inhibitor H-89 (10 μM) restored T (Fig. 3a) and E_2_ (Fig. 3b) contents to levels comparable to those of the solvent control (*P* > 0.05).

### The target site of action of BPS is the cell membrane

**Fig. 4:**
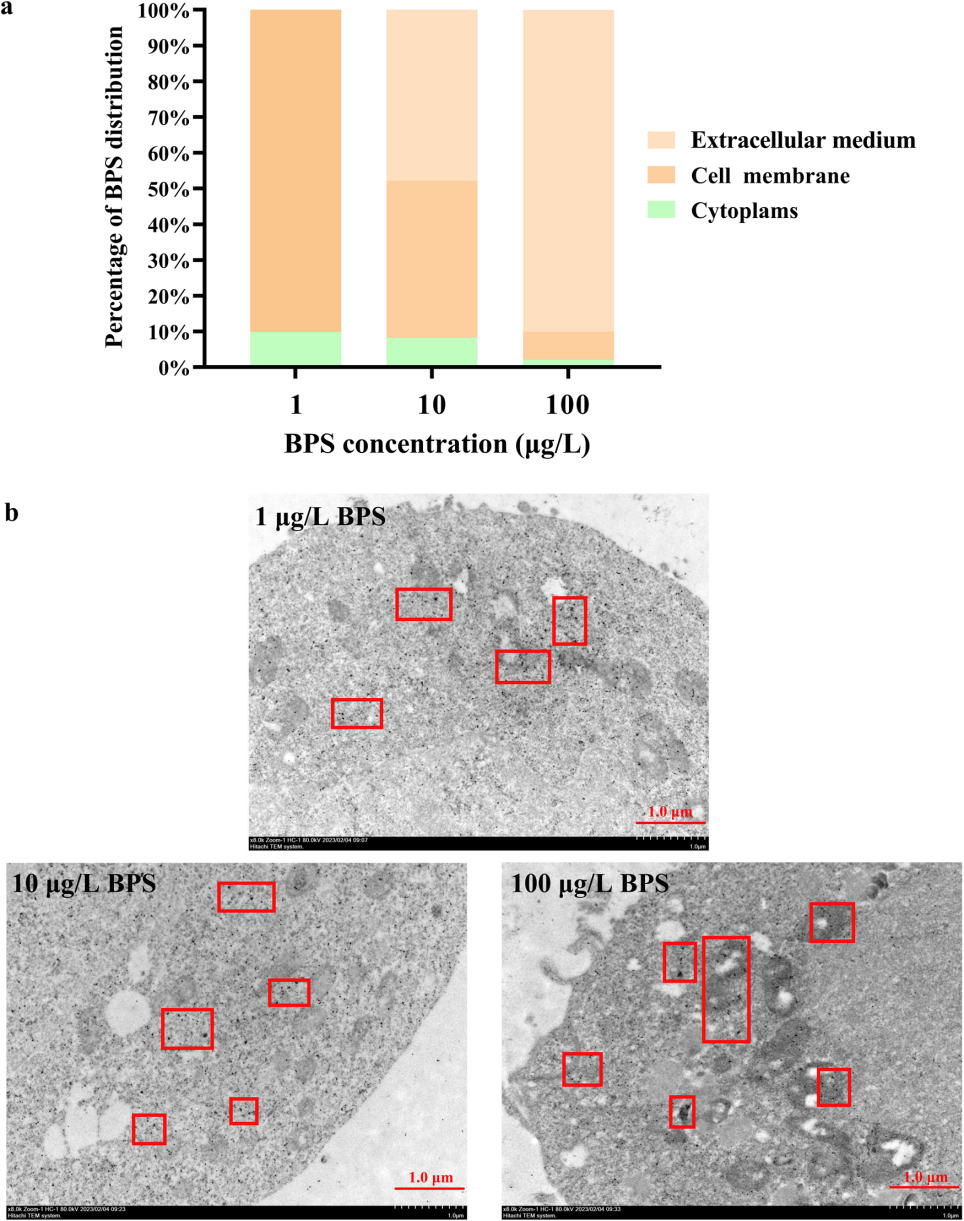
BPS accumulated mainly on the cell membrane in SVOG cells after exposure for 48 h. **a** Subcellular distribution of BPS quantified by LC-MS/MS. **b** Representative images of immunoelectron microscopy. The red square highlights gold particles, and the scale bar is 1 μm.

The results of liquid chromatography-tandem mass spectrometry (LC-MS/MS) showed that after exposure to BPS at nominal concentrations of 1 μg/L, 10 μg/L and 100 μg/L for 48 h, 0%, 47.83%, and 90.02% were retained in the extracellular medium, respectively, and 100%, 52.17% and 9.98% of the BPS was accumulated by human ovarian granulosa cells SVOG, respectively (Fig. 4a). Approximately 80%-90% of accumulated BPS was absorbed on the cell membrane, while approximately 10%-20% entered in the cytoplasm (Fig. 4a). According to the results of immunoelectron microscopy, gold particles were observed in BPS treatments (Fig. 4b).

**Fig. 5:**
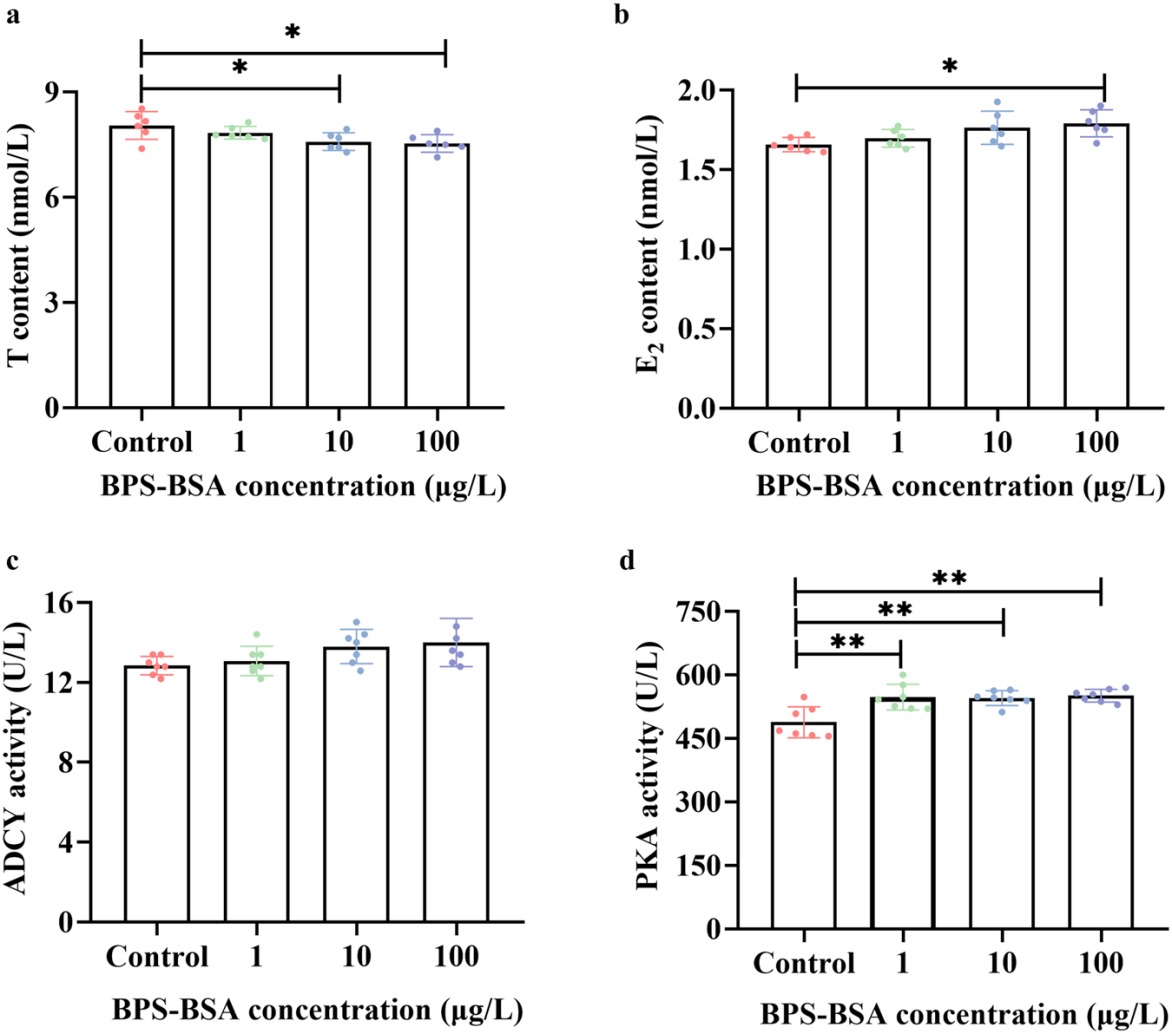
Impermeable BPS-BSA exposure for 48 h still caused excessive estrogen synthesis and upregulated cAMP/PKA signaling pathway in SVOG cells. **a** T content. *n* = 6. **b** E_2_ content. *n* = 6. **c** ADCY activity. *n* = 7. **d** PKA activity. *n* = 7. Data are expressed as mean ± SD. * indicated a significant difference from the solvent control (0.01 < *P* < 0.05), and ** indicated a highly significant difference from the solvent control (*P* < 0.01).

Furthermore, impermeable BPS-BSA at equivalent BPS concentrations of 10 μg/L and 100 μg/L still downregulated T content (0.01 < *P* < 0.05) (Fig. 5a), and BPS-BSA at equivalent BPS concentration of 100 μg/L still upregulated E_2_ content (0.01 < *P* < 0.05) (Fig. 5b). BPS-BSA exposure at equivalent BPS concentrations of 1 μg/L, 10 μg/L, and 100 μg/L still upregulated PKA activities (*P* < 0.01) (Fig. 5d), while ADCY activities were not influenced (*P* > 0.05) (Fig. 5c).

Under the condition of supplementation with T, E_2_ content was still upregulated by BPS-BSA at equivalent BPS concentration of 100 μg/L (*P* < 0.01) (Fig. S2b).

### The target molecule of action of BPS is FSHR located on the cell membrane

**Fig. 6:**
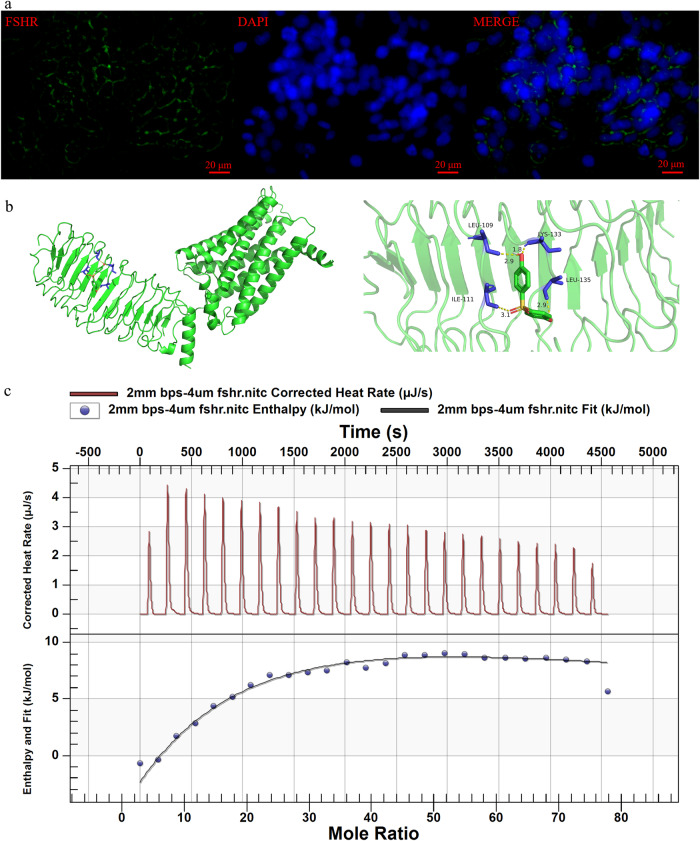
Interaction of BPS with FSHR. **a** Representative images of immunofluorescence staining for subcellular localization of FSHR. **b** Binding mode simulated by molecular docking. **c** Calorimetric map by isothermal titration calorimetry analysis. The top panel, the corrected heat rate of each titration of BPS into FSHR against time; the bottom panel, integrated heat data in terms of enthalpy plotted against mole ratio of BPS/FSHR.

According to the results of immunofluorescence, FSHR was selectively expressed on the cell membrane of SVOG cells (Fig. 6a), and FSHR expression was not affected by BPS exposure (Fig. S4).

The results of molecular docking showed that BPS was docked into extracellular domain of FSHR *via* formation of hydrogen bonds (Fig. 6b). To be specific, one of the two hydroxyl groups of BPS formed two hydrogen bonds with LEU-109 and LYS-133 of FSHR, the other hydroxyl group formed one hydrogen bond with IEU-135 of FSHR, and the sulfone group formed one hydrogen bond with ILE-111 of FSHR (Fig. 6b).

Based on the calorimetric map of the interaction between BPS and FSHR by isothermal titration calorimetry analysis (Fig. 6c), thermodynamic parameters were obtained by the calculation using “sequential binding sites” model (Table 1). The most matched numbers of binding sites are two (Table 1). For binding site 1, the binding constant Kd was determined to be 1.83 × 10^-4 ^M, enthalpy *ΔH* was −1.69 × 10^2^ KJ mol^−1^, and entropy *ΔS* was −4.94 × 10^2 ^J mol^−1^ K^−1^ (Table 1). For binding site 2, the binding constant Kd was determined to be 1.09 × 10^−3 ^M, enthalpy *ΔH* was 5.00 × 10^3^ KJ mol^−1^, and entropy *ΔS* was 1.68 × 10^4 ^J mol^−1^ K^−1^ (Table 1).

**Table 1:** Thermodynamic parameters of the interaction between BPS and FSHR by isothermal titration calorimetry analysis

Parameter	Value for specific binding site	
*n*	*n*_1_	*n*_2_
Kd (M)	1.83 × 10^−4^	1.09 × 10^−3^
Ka (M^−1^)	5.47 × 10^3^	9.17 × 10^2^
*ΔH* (KJ mol^−1^)	−1.69 × 10^2^	5.00 × 10^3^
*ΔS* (J mol^−1^ K^−1^)	−4.94 × 10^2^	1.68 × 10^4^

**Fig. 7:**
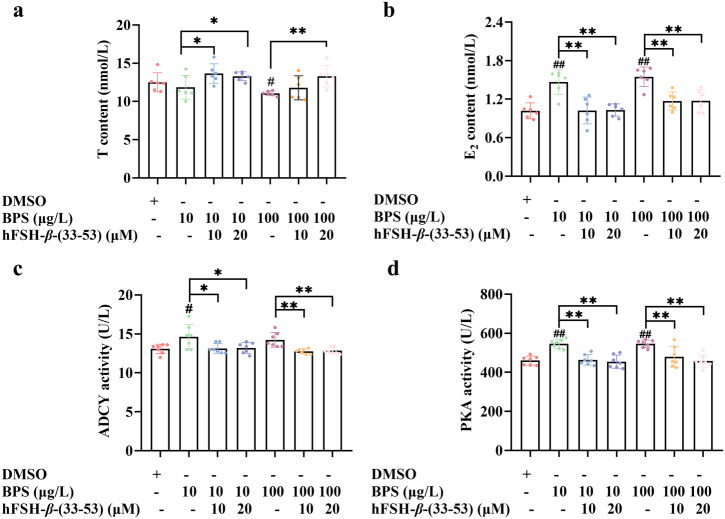
Combined exposure to BPS and FSHR antagonist hFSH-*β*-(33-53) restored estrogen synthesis and cAMP/PKA signaling pathway to levels comparable to those of the solvent control in SVOG cells. **a** T content. *n* = 6. **b** E_2_ content. *n* = 6. **c** ADCY activity. *n* = 7. **d** PKA activity. n = 7. Data are expressed as mean ± SD. ^#^ indicated a significant difference from the solvent control (0.01 < *P* < 0.05), and ^##^ indicated a highly significant difference from the solvent control (0.01 < *P* < 0.05). * indicated a significant difference between the combined exposure group to BPS and hFSH-*β*-(33-53) and the BPS single exposure group *(*0.01 < *P* < 0.05), and ** indicated a highly significant difference between the combined exposure group to BPS and hFSH-*β*-(33-53) and the BPS single exposure group (*P* < 0.01).

Furthermore, combined exposure to BPS (10 μg/L and 100 μg/L) and FSHR antagonist hFSH-*β*-(33-53) (10 μM and 20 μM) restored T content (Fig. 7a), E_2_ content (Fig. 7b), ADCY activities (Fig. 7c) and PKA activities (Fig. 7d) to levels comparable to those of the solvent control (*P* > 0.05).

## Discussion

Ovarian steroidogenesis is complex because the enzymatic steps are partitioned between the granulosa and theca cells, which surround the oocyte and form a follicle. The endoplasmic reticulum of granulosa cells contains abundant aromatase CYP19A1, catalyzing the conversion of T to E_2_, which is considered as the rate-limiting step in E_2_ biosynthesis^1,21,22^. In this study, we found that *CYP19A1* gene expression, CYP19A1 protein expression and CYP19A1 enzyme activities and E_2_/T ratio were upregulated in human ovarian granulosa cells SVOG after BPS exposure, with inverse U-shaped dose-response relationships observed for CYP19A1 enzyme activities and *CYP19A1* gene expression. In fact, the non-monotonic dose response is thought to be a general phenomenon for EDCs^26,27^_._ Our findings are consistent with previous in vivo and in vitro studies demonstrating that BPS exposure causes excessive estrogen synthesis^3–9^. For example, serum E_2_ levels were increased in CD-1 mouse injected subcutaneously with BPS (50 μg/kg or 10 mg/kg) every three days from birth to postnatal day 60^3^, and treatment of ovine granulosa cells with BPS at concentrations ranging from 10 µM (2.50 mg/L) to 200 µM (50.05 mg/L) for 48 h increased E_2_ secretion^8^. However, human epidemiological evidence is still needed to validate the effects of BPS exposure on hormone homeostasis in women^27,28^. Besides, special attention should be paid to gender differences when exploring the effects of BPS on sex hormone homeostasis. Although animal experimental studies have provided compelling evidence that BPS exposure also led to excessive levels of estrogen in males^3,5–7^, human epidemiological investigations showed that BPS exposures were negatively associated with serum E_2_ and E_2_/T ratio in males^28,29^.

cAMP/PKA pathway is considered to be the primary signaling cascade mediating estrogen biosynthesis^23–25^. An elevation of intracellular cAMP is activated by ADCY^23–25^. cAMP serves as a second messenger, which in turn activates PKA that phosphorylates CREB^23–25^. Phosphorylated CREB (p-CREB) bind to the cAMP-responsive element (CRE) in the promoter region of the *CYP19A1* gene, thus promoting *CYP19A1* gene transcription^23–25^. Our results showed that all of these indicators were significantly upregulated by BPS exposure. Furthermore, the inhibitory effects of BPS on T content and stimulatory effects on E_2_ levels were blocked by PKA inhibitor H-89^30^, suggesting that BPS causes excessive estrogen synthesis in human ovarian granulosa cells through the cAMP/PKA signaling pathway.

Transmission of various extracellular signals into intracellular responses mediated by signaling cascades is triggered by conformational changes of receptors located on the cell membrane^31^. Our results of LC-MS/MS showed that the majority of bioaccumulated BPS (approximately 80%-90%) was absorbed on the cell membrane, with only a minority (approximately 10%-20%) entering the cytoplasm. Consistent with our findings for BPS, Fei et al.^32^ found that after 8 h of exposure BPA remained free in the medium, absorbed on the cell membrane and entered the cytoplasm was about 90%, 10%, and 1%, respectively. Cell membranes is a bilayer matrix primarily composed of phospholipids and embedded by proteins^33^. Each phospholipid consists of a hydrophilic phosphate head group linked to one or two hydrophobic long-chain fatty acid tail(s), where the head faces aqueous cytosol and extracellular fluid and the tail(s) face toward the inner area of the membrane bilayer^33^. The transport of contaminants into cells mainly depends on hydrophobic interactions^32^. We suggest that due to the two hydrophilic hydroxyl groups and the hydrophilic sulfone group, BPS is not prone to penetrate the phospholipid bilayer and enter the cell interior. Although the toxicological effects of BPS have been elucidated by many studies, and the distribution of BPS and its metabolites in major internal organs has been examined in animal experiments^34,35^, its transmembrane transport has received little attention. To our knowledge, this is the first time that the subcellular distribution of BPS has been detected.

The results of immunoelectron microscopy confirmed the small accumulation of BPS in the cytoplasm. Thus, BPS–BSA complex exposure experiment was employed to clarify that the site of action of BPS is the cell membrane. BPS is a small molecule chemical with a relative molecular weight of 250.27^36^, and BSA is a selective macromolecular carrier with a molecular weight of 66.446 kDa^37,38^, making BPS-BSA an impermeable macromolecular complex^37,38^. The results showed that BPS-BSA, like unconjugated BPS, still caused excessive estrogen synthesis and upregulated cAMP/PKA signaling pathway. Since the BPS-BSA complex cannot penetrate the cell membrane and enter the cell interior, it can be assumed that the stimulatory effects on estrogen synthesis by BPS are non-genomic effects caused by interaction with certain target molecule located on the cell membrane, rather than genomic effects caused by interaction with any nuclear receptors inside the cell. Similarly, the impeded ligand of BSA has already been used in several studies to verify the existence of the non-genomic mechanism of action of endogenous hormones and exogenous EDCs^37,38^. For example, E_2_-BSA conjugate, as did the unconjugated E_2_, could act *via* a membrane version of the estrogen receptor *α* on pituitary tumor cells and breast cancer cells and provoke Ca^2+^ influx *via* L-type channels, leading to prolactin secretion, suggesting the existence of the non-genomic responses mediated by membrane estrogen receptor *α*^37^.

Membrane receptors are important components of cell membranes and also undertake primary functions for cell membranes^31^. Unlike the “slow” genomic effects mediated by nuclear receptors, the “rapid” cellular responses mediated by membrane receptors are called “non-genomic” effects, which play an important role in the process leading to the adverse effects caused by EDC exposure^39^. By using a combination of immunofluorescence, molecular docking, isothermal titration calorimetry and combined exposure of BPS and FSHR antagonist, it was demonstrated that FSHR located on the cell membrane is the molecule of action of BPS.

FSHR displays a high degree of tissue specificity as in females, it is only expressed in granulosa cells^25^. As a member of the G protein-coupled receptor (GPCR) superfamily, FSHR belongs to the glycoprotein hormone receptor (GPHR) cluster within the rhodopsin family, and it is characterized by a large extracellular domain (ECD), seven transmembrane helices, and a short intracellular C-terminal tail^23,40^. FSHR binds its natural ligand, FSH, through its characteristic large horse-shoe-shaped ECD. The ECD of FSHR contains 12 LEU/ILE-rich repeat (LRR) sequences, and the interaction of FSH with LRR1-9 leads to conformational rearrangements within the transmembrane regions, thereby activating the complex intracellular signaling cascades^25,41^. Our results of immunofluorescence showed that FSHR was selectively expressed on the cell membrane, which is consistent with the observation of Hanyroup et al.^42^. The results of molecular docking showed that BPS was docked into extracellular domain of FSHR *via* formation of hydrogen bonds with amino acids in LRR sequences, including LEU-109 and ILE-111 in the LRR4 motif and LYS-133 and IEU-135 in the LRR5 motif. Consistent with the results of molecular docking, isothermal titration calorimetry analysis confirmed the direct interaction between BPS and FSHR. Furthermore, synthetic peptide hFSH-*β*-(33-53), a specific FSHR binding inhibitor (antagonist)^43^, blocked BPS-stimulated estrogen synthesis and cAMP/PKA signaling pathway, suggesting that transmission of extracellular BPS signal into intracellular responses (estrogen synthesis) mediated by cAMP/PKA signaling pathway is triggered by interaction of BPS with FSHR located on the cell membrane.

FSHR inactivating mutations may cause amenorrhea, infertility, and premature ovarian failure, whereas activating mutations can predispose to ovarian hyperstimulation syndrome, implying the importance of the FSHR function in female reproduction^23^. To date, depending on binding sites and the effects on downstream pathways, several classes of small molecule ligands have been identified as FSHR orthosteric agonists, orthosteric antagonists, allosteric agonists and allosteric antagonists^40,44^. Agonists include diketopiperazines, hexahydroquinolines, thiazolidinones, and thienopyrimidines, and antagonists include benzamide derivatives, (bis)benzamides, sulfonic acid, (bis)sulfonic acid and tetrahydroquinolines^40,44^. Since the binding sites of BPS overlap with that of the natural ligand, and cAMP/PKA signaling pathway was upregulated upon BPS exposure, we consider BPS as an FSHR orthosteric agonist. Interestingly, consistent with our findings for BPS, other FSHR orthosteric modulators also exhibit selective signaling profiles (a phenomenon referred to as “biased-signaling”)^25,44^. Besides, these small molecules as agonists are proposed to have somehow changed the receptor form to expose more FSH-binding sites^41^.

There were a few limitations in this study. For example, although the effects of BPS on androgen-stimulated estrogen production were investigated in human ovarian granulosa cells in vitro models, no supplementation with FSH was performed. On one side, considering the “two cell, two gonadotropin theory” of steroid hormone production in the ovary^21,22^, the steroidogenic ability of human ovarian granulosa cells could not be fully reflected, and this may explain the overall small effect size observed. One the other side, by performing experiments of combined exposure to BPS and FSHR antagonist, our study demonstrated that BPS could agonize FSHR without concomitant FSH supplementation, but it is still unknown whether BPS would compete with FSH for binding FSHR when receptor is not enough for its ligands due to FSH supplementation, especially at high concentrations. Zhang et al.^45^ reported thyroid hormone signaling disruption of bisphenols in a biphasic manner.

**Fig. 8:**
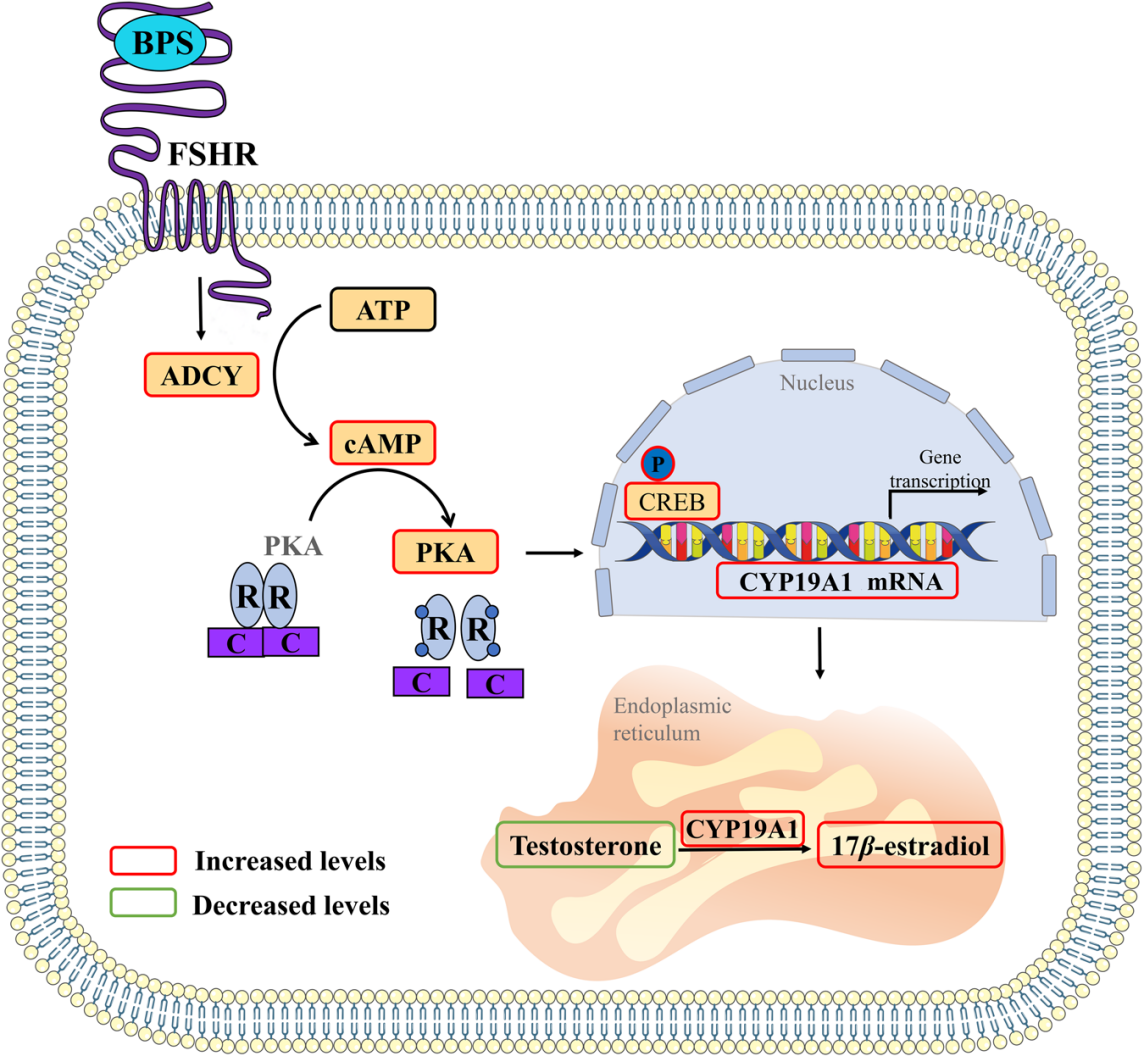
Schematic illustrates the underlying signaling pathway-mediated mechanisms, target site and target molecule of action of BPS causing excessive estrogen synthesis. BPS accumulates mainly on the cell membrane of SVOG cells, binds to FSHR located on the cell membrane, and subsequently activates the downstream cAMP/PKA signaling pathway, leading to enhanced conversion of T to E_2_.

The results of this study confirms that BPS accumulates mainly on the cell membrane, binds to FSHR located on the cell membrane, and subsequently activates the downstream cAMP/PKA signaling pathway, leading to enhanced conversion of T to E_2_ (Fig. 8). This study deepens our knowledge of the mechanisms of environmental factors in pathogenesis of hyperestrogenism, which is indispensable for the prevention and treatment of hyperestrogenism and a diverse array of hyper-estrogen activity-driven diseases.

## Materials and methods

### Chemicals

BPS (≥98% purity, CAS No: 80-09-1) was purchased from Sigma-Aldrich (St. Louis, MO, USA). BPS was dissolved in dimethyl sulfoxide (DMSO, ≥99.5% purity, CAS No: 67-68-5, Solarbio, Beijing, China) to prepare stock solutions. The stock solutions were stored at room temperature, protected from light, and renewed every two days. The final concentration of DMSO did not exceed 0.1% in all experiments.

BPS-BSA coupling (30: 1) using methyl 4-bromobutyrate as the linker arm was obtained by dialysis and sterilization, and verified using sodium dodecyl sulfate-polyacrylamide gel electrophoresis (SDS-PAGE) and Fourier transform infrared spectroscopy (FTIR).

### Cell culture and chemical exposure

Nontumorigenic immortalized human granulosa cells (SVOG) were purchased from Qingqi Biotechnology (Shanghai, China), seeded in 6-well plates at a density of 1.5 × 10^5^ cells/mL and routinely maintained in standard Dulbecco’s modified eagle medium (DMEM, VivaCell, Shanghai, China) supplemented with 10% fetal bovine serum (Biological Industries, Kibbutz Beit-Haemek, Israel), 2 mM L-glutamine, 100 μg/mL streptomycin and 100 U/mL penicillin (all obtained from Solarbio, Beijing, China) at 37 °C with 5% CO_2_ under saturated humidity. To avoid interference by endogenous hormones, cells at the logarithmic growth stage were kept in phenol-red-free DMEM (VivaCell, Shanghai, China) supplemented with 10% hormone-free fetal bovine serum (Biological Industries, Kibbutz Beit-Haemek, Israel) for chemical exposure experiments.

SVOG cells were exposed to 0 μg/L (DMSO solvent control), 1 μg/L, 10 μg/L and 100 μg/L BPS for 48 h. The actual concentrations of BPS in the extracellular medium were detected by liquid chromatography-tandem mass spectrometry (LC-MS/MS) at the beginning of the exposure test, which were maintained within the range of 97% - 120% of the nominal concentrations. All results are shown according to nominal BPS concentrations. The levels of BPS in samples of the solvent control group were below the detection limit. Cell viability was determined based on the measured absorbance at 450 nm using a microplate reader (Synergy LX, BioTek, Winooski, VT, USA) after treating with Cell Counting Kit-8 (CCK-8) (Beyotime, Shanghai, China). After 48 h of exposure, the extracellular medium was collected for ELISA and LC-MS/MS analyses. Cells were sampled for immunofluorescence. Or, after the cells were digested with 0.25% trypsin-EDTA (Solarbio, Beijing, China), dispersed from clumps into single cells, and centrifuged at 1000 r/min for 10 min, the supernatant (cell eluent) was collected for LC-MS/MS analysis. Cells were sampled for real-time polymerase chain reaction (PCR) assays, lysed in RIPA lysis buffer (Solarbio, Beijing, China) for further analyses of ELISA, Western blotting and LC-MS/MS, fixed in 2.5% glutaraldehyde fixative (Beyotime, Shanghai, China) for transmission electron microscopy observation, or fixed in fixative for immunoelectron microscopy (Servicebio, Wuhan, China).

After 48 h of exposure to 1 μg/L, 10 μg/L and 100 μg/L BPS under the condition of supplementation with 50 nmol/L T (≥ 98% purity, CAS No: 58-22-0, Solarbio, Beijing, China), the extracellular medium was collected for ELISA.

After 48 h of exposure to 10 μg/L and 100 μg/L BPS in combination with 10 μM PKA inhibitor H-89 (Beyotime, Shanghai, China), the extracellular medium was collected for ELISA.

After 48 h of exposure to BPS-BSA with equivalent BPS concentrations of 1 μg/L, 10 μg/L, and 100 μg/L, the extracellular medium was collected for ELISA, and cells were lysed in RIPA lysis buffer for further analyses of ELISA.

After 48 h of exposure to BPS-BSA with equivalent BPS concentrations of 1 μg/L, 10 μg/L, and 100 μg/L under the condition of supplementation with 50 nmol/L T, the extracellular medium was collected for ELISA.

After 48 h of exposure to 10 μg/L and 100 μg/L BPS in combination with 10 μM and 20 μM FSHR antagonist hFSH-*β*-(33-53) (MCE, Shanghai, China), the extracellular medium was collected for ELISA, and cells were lysed in RIPA lysis buffer for further analyses of ELISA.

### Real-time quantitative PCR

The mRNA levels of *CYP19A1* and *ADCY* were measured by real-time quantitative PCR. Total RNA was extracted with Trizol (Vazyme, Nanjing, China). Reverse transcription was performed with HiScript^®^ III RT SuperMix for qPCR ( + gDNA wiper) (Vazyme, Nanjing, China). Real-time PCR was performed using Taq Pro Universal SYBR qPCR Master Mix (Vazyme, Nanjing, China) with a Mastercycler ep RealPlex (Eppendrop, Wesseling-Berzdorf, Germany). Primer sequences designed by Primer Premier 6.0 software (PREMIER Biosoft International, Palo Alto, CA, USA) are shown in Table S1. The reaction conditions were as follows: 95 °C pre-denaturation for 30 s, followed by 40 cycles of 95 °C for 5 s, 60 °C for 30 s, and 72 °C for 30 s, and then melting curve analysis was performed. *GAPDH* was used as internal standard, and fold changes in gene expression were calculated using the 2^-∆∆Ct^ method.

### ELISA

The concentrations of T and E_2_ in the extracellular medium, intracellular cAMP concentrations, and intracellular PKA, PKC, and ADCY activities were detected using ELISA kits from Mlbio (Shanghai, China). The intracellular CYP19A1 activities, CREB concentrations and p-CREB concentrations were detected using ELISA kits from Meimian (Yancheng, China). E_2_/T ratio was calculated, and CREB phosphorylation was presented as the ratio between p-CREB concentration and CREB concentration. The inter-assay and intra-assay variation coefficients of all kits were <10%.

### Western blotting

The expression levels of CREB, p-CREB, ADCY, and CYP11A1 were also detected using Western blotting. The cell lysate was electrophoresed on 10% SDS-PAGE gel and then transferred onto polyvinylidene fluoride membrane (MerckMillipore, Darmstadt, Germany). The membrane was blocked with 5% nonfat dry milk solution, incubated with primary antibodies (Phospho-CREB (Ser133) (87G3) Rabbit mAb, CST, Danvers, MA, USA, 1:500, CREB (48H2) Rabbit mAb, CST, Danvers, MA, USA, 1:500, ADCY Rabbit pAb, Abclonal, Wuhan, China, 1:1000, CYP11A1 Rabbit mAb, Abclonal, Wuhan, China, 1:1000 or GAPDH Rabbit pAb, Abbkine, Wuhan, China, 1:10,000) at 4 °C overnight, and incubated with secondary antibody (HRP Goat Anti-Rabbit lgG, Abbkine, Wuhan, China, 1:10,000) at room temperature for 1 h. Immunoblot membrane was developed with Ultrasensitive ECL Chemiluminescence Kit (Beyotime, Shanghai, China). The results were detected by a Tanon 5200 chemiluminescence/fluorescence image analysis system (Tanon, Shanghai, China). The optical densities were expressed in arbitrary units determined by Image J 1.8.0 (NIH, Washington, DC, USA). GAPDH served as a loading control.

### LC-MS/MS

The actual concentrations of BPS in the extracellular medium, cell eluent, and cell lysate, representing extracellular, membrane, and cytoplasmic distribution, respectively, were quantified by LC-MS/MS using an ultra-performance liquid chromatography system (Thermo Fisher Scientific, San Jose, CA, USA) coupled to a Q-Exactive tandem mass spectrometer (Thermo Fisher Scientific, San Jose, CA, USA). The samples were spiked with the internal standard D_16_-BPA (≥ 97.9% purity, Sigma-Aldrich, St. Louis, MO, USA), enzymatically digested by *β*-glucuronidase (100 U, Sigma-Aldrich, St. Louis, MO, USA) in ammonium acetate buffer (pH = 5.0) at 37 °C overnight, and extracted with methanol for 5 h. After centrifugation at 3000 r/min for 15 min, the supernatants were collected and transferred to vials with inserts for instrumental analysis. Chromatographic separation was performed on an ACQUITY UPLC BEH C_18_ (2.1 × 150 mm, 1.7 μm particle size) column (Waters, Milford, MA, USA). The mobile phases consisted of methanol (solvent A) and 2 mM ammonium formate in water (solvent B), and the flow rate was 0.4 mL/min. The mass spectrometer was performed with negative electron spray ionization mode and operated in multiple reaction monitoring modes. The regression coefficient (R^2^) of the calibration curve was >0.99. The limit of detection of BPS was 0.0015 ng/mL. No background interference was observed.

### Electron microscopic observation

#### Transmission electron microscopy

Glutaraldehyde-fixed cells were gently scraped with a cell scraper and harvested by centrifugation at 3000 r/min for 2 min. Following fixation in 1% osmium tetroxide (Ted Pella Inc, Redding, CA, USA), dehydration in a series of graded ethanol and anhydrous acetone, embedding in 812 embedding agent (SPI, Beijing, China), ultrathin sections (60–80 nm) were obtained with a Leica EM UC7 Ultramicrotome (Leica, Wetzlar, Germany). The section was stained with 2% uranyl acetate and 2.6% lead citrate, and observed under a HT7800 transmission electron microscope (Hitachi, Tokyo, Japan). The width of endoplasmic reticulum was measured using Image J 1.8.0 (NIH, Washington, DC, USA) according to the method proposed by Lam et al.^46^.

#### Immunoelectron microscopy

Cells fixed in fixative for immunoelectron microscopy were gently scraped with a cell scraper and harvested by centrifugation at 1000 r/min for 5 min. Following dehydration in a series of graded ethanol and embedding in resin, ultrathin sections (60–80 nm) were obtained with a Leica EM UC7 Ultramicrotome (Leica, Wetzlar, Germany). Non-specific binding was blocked with 1% BSA in Tris buffered saline, incubated with primary antibody (BPS mouse monoclonal antibody, prepared in our laboratory using the hybridoma technique with the BPS-BSA complex as the complete antigen, 1:200) at 4 °C overnight, and incubated with secondary antibody (10 nm Colloidal Gold Labeled Goat Anti-mouse lgG, Sigma-Aldrich, St. Louis, MO, USA, 1:50) at 37 °C for 1 h. Gold particles were observed under a HT7800 transmission electron microscope (Hitachi, Tokyo, Japan).

### Immunofluorescence

The subcellular localization and relative expression of FSHR were detected by immunofluorescence. Cells were immersed sequentially in 4% paraformaldehyde (Sint-bio, Shanghai, China) for 10 min, 0.2% triton X-100 (Beyotime, Shanghai, China) for 10 min, and 5% BSA for 1 h. After overnight incubation at 4°C with primary antibodies (FSHR rabbit polyclonal antibody, 1:200, ABclonal, Wuhan, China) and then 1 h incubation at room temperature with species-specific secondary antibodies (goat anti-rabbit IgG (H&L)-Alexa Fluor 488, 1:500, ABclonal, Wuhan, China), nuclei were stained with 4’,6-diamidino-2-phenylindole (DAPI, Solarbio, Beijing, China) for 10 min at room temperature. After each step of immunofluorescence procedure, cells were carefully rinsed with phosphate buffer saline (PBS) containing 0.1% Tween-20. After samples were mounted with mounting medium, images were captured using an Eclipse Ti2 fluorescence microscopy (Nikon, Tokyo, Japan) and digitally processed using Image J 1.8.0 (NIH, Washington, DC, USA).

### Molecular docking

Molecular docking was employed to predict the binding potential and binding sites of BPS with FSHR. The X-ray structure of human FSHR (PDB entry: 8I2H) obtained from the RCSB Protein Data Bank (https://www.rcsb.org/structure) was used as input after eliminating water molecules using the PyMOL software (http://www.pymol.org). The 2D sdf format file of BPS (Compound CID: 6626) was downloaded from the National Center for Biotechnology Information (https://pubchem.ncbi.nlm.nih.gov) and converted to a 3D pdb format structure using OpenBabelGUI 2.4.1 (http://openbabel.org/wiki/Main_Page). BPS was docked into human FSHR using Auto Dock 4.2 (Scripps Research Institute, La Jolla, CA, USA). The optimal conformation with the lowest binding energy (−4.20 kcal mol^−1^) was chosen to visually analyze the binding mode through the application of PyMOL software (http://www.pymol.org).

### Isothermal titration calorimetry

Isothermal titration calorimetry was conducted to verify the direct interaction between BPS and FSHR obtained from molecular docking simulations. BPS was dissolved in PBS solution (containing 5% DMSO) at a final concentration of 2 mM with a final volume of 50.5 μL, and FSHR (Huamei, Wuhan, China) was dissolved in PBS solution (containing 5% DMSO) at a final concentration of 4 μM with a final volume of 350 μL. FSHR was titrated with BPS using a Nano ITC (TA Instruments, New Castle, DE, USA) with titration conditions as follows: number of injection, 25; volume per injection, 2.02 μL; injection interval, 180 s; temperature, 25 °C and stirring rate, 350 r/min.

### Statistical analysis

All experiments were repeated at least three times and data are expressed as mean ± standard deviation. Statistical differences between groups were determined using one-way analysis of variance (ANOVA) followed by Tukey’s post hoc testing. Differences were considered significant when 0.01 < *P* < 0.05, and differences were considered highly significant when *P* < 0.01.

### Supplementary information

Supporting Information

Description of additional supplementary files

Supplementary data

## Data Availability

All data supporting the findings of this study are available within the article and its Supplementary Information. Unprocessed blots are presented in Supplementary Fig. S5. All source data underlying the graphs and charts presented in the main figures are available in the Supplementary Data file. All other data are available from the corresponding author upon reasonable request.
