# Supplementary material for: Bisphenol S causes excessive estrogen synthesis by activating FSHR and the downstream cAMP/PKA signaling pathway
Source: Commun Biol. 2024 Jul 10;7:844. doi: 10.1038/s42003-024-06449-2 (PMC11237073; doi:10.1038/s42003-024-06449-2)
Supplement: Supplementary file 1 — Supporting Information [file 42003_2024_6449_MOESM1_ESM.pdf]

Supporting Information

Bisphenol S causes excessive estrogen synthesis by activating FSHR and the downstream cAMP/PKA signaling pathway

Authors: Xiaorong Zhang[a,b,#], Xinda Zhang[a,#], Zhenzhong Zhang[a], Yijiao Shi[a], Jun Wang[a], Shaoguo Ru[a], Hua Tian[a,*]

[a]College of Marine Life Sciences, Ocean University of China, Qingdao 266003, Shandong Province, China

[b]Tai'an Agriculture and Rural Affairs Bureau, Tai'an 271000, Shandong Province, China

Page #

Fig. S1. No significant cytotoxicity was observed in SVOG cells after BPS exposure for 48 h. S2

Fig. S2. Exposure to BPS (a) and BPS-BSA (b) for 48 h under the condition of supplementation with T caused excessive estrogen synthesis in SVOG cells. S3

Fig. S3. No significant effect on PKC activities was observed in SVOG cells after BPS exposure for 48 h. S4

Fig. S4. No significant effect on FSHR expression was observed in SVOG cells after BPS exposure for 48 h. S5

Fig. S5. Unprocessed blots. S6

Table S1. Primer sequences. S7

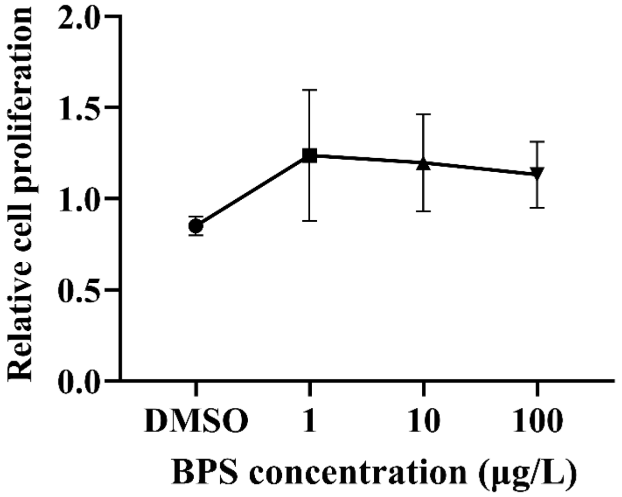

Fig. S1. No significant cytotoxicity was observed in SVOG cells after BPS exposure for 48 h. n = 9.

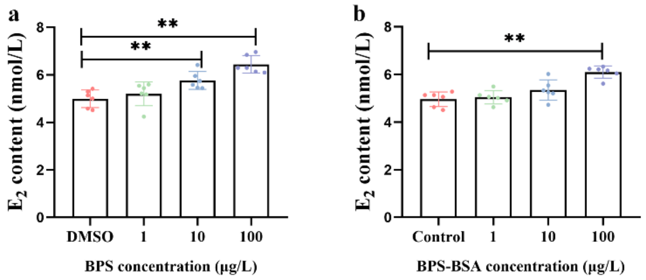

Fig. S2. Exposure to BPS (a) and BPS-BSA (b) for 48 h under the condition of supplementation with T caused excessive estrogen synthesis in SVOG cells. n = 6. Data are expressed as mean ± SD. ** indicated a highly significant difference from the solvent control ($P < 0.01$).

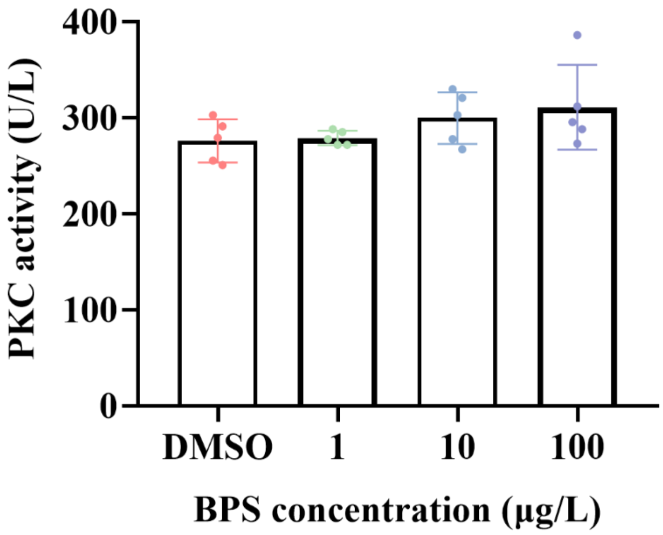

Fig. S3. No significant effect on PKC activities was observed in SVOG cells after BPS exposure for 48 h. n = 5.

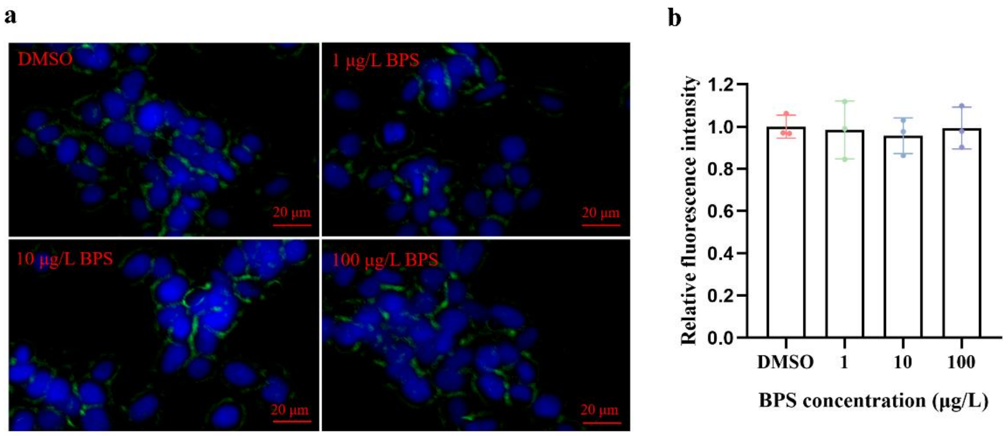

Fig. S4. No significant effect on FSHR expression was observed in SVOG cells after BPS exposure for 48 h. a Representative images of immunofluorescence staining for relative expression of FSHR. **b** Densitometric analysis of immunofluorescence for relative expression of FSHR. n = 3.

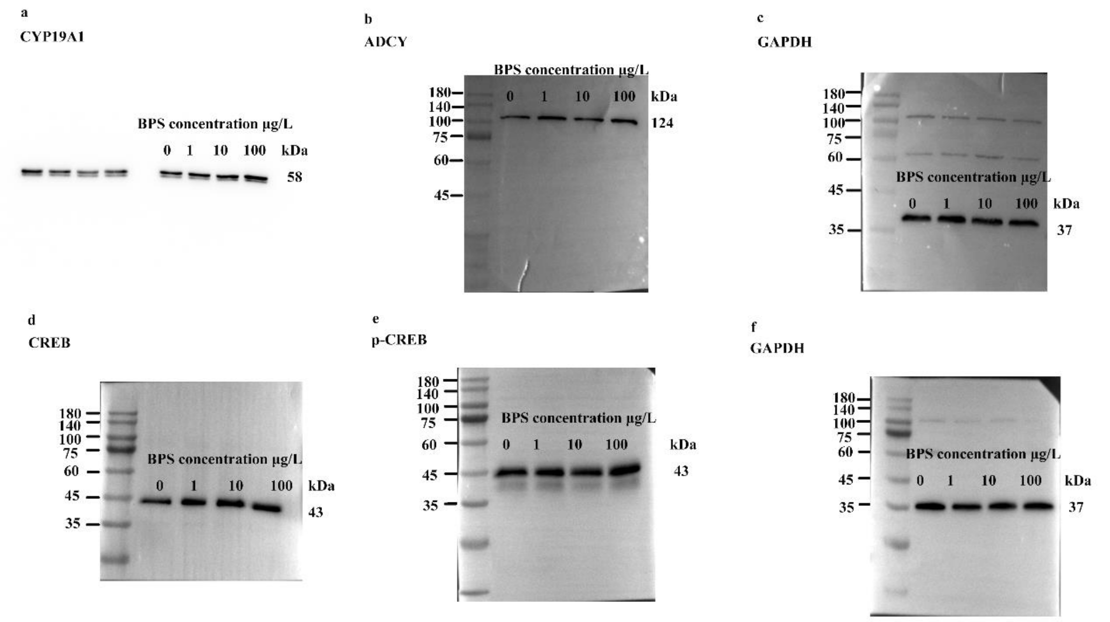

Fig. S5. Unprocessed blots. a CYP19A1 blots for Figure 1e. **b** ADCY blots for Figure 2c. **c** GAPDH blots for Figure 1e and 2c. **d** CREB blots for Figure 2g. **e** p-CREB blots for Figure 2g. **f** GAPDH blots for Figure 2g.

Table S1. Primer sequences.

Gene	Primer sequence (5′-3′)	GenBank Accession No.
CYP19A1	F: CACAGCGAGGAAGGAGGAGAGG	NM_000103.4
	R: TGGCTTAGGAACCTGGAGAGATGG	
ADCY	F: GGGAGAGAAAGGGAAACGATGTCAG	NM_020546.3
	R: AAGCAACGAAGCACAGGGAGATG	
GAPDH	F: GCACCGTCAAGGCTGAGAAC	NM_001256799.3
	R: TGGTGAAGACGCCAGGGA	
