# Supplementary material for: Bisphenol S causes excessive estrogen synthesis by activating FSHR and the downstream cAMP/PKA signaling pathway
Source: Commun Biol. 2024 Jul 10;7:844. doi: 10.1038/s42003-024-06449-2 (PMC11237073; doi:10.1038/s42003-024-06449-2)
Supplement: Supplementary file 2 — Description of additional supplementary files [file 42003_2024_6449_MOESM2_ESM.pdf]

Description of Additional Supplementary Files

File name: Supplementary Data

Description: Source data underlying the graphs and charts presented in the main figures
